# Coral-Like Yolk–Shell-Structured Nickel Oxide/Carbon Composite Microspheres for High-Performance Li-Ion Storage Anodes

**DOI:** 10.1007/s40820-018-0234-0

**Published:** 2019-01-09

**Authors:** Min Su Jo, Subrata Ghosh, Sang Mun Jeong, Yun Chan Kang, Jung Sang Cho

**Affiliations:** 1 Department of Engineering Chemistry, Chungbuk National University, Chungbuk, 361-763 Republic of Korea; 2 Department of Chemical Engineering, Chungbuk National University, Chungbuk, 361-763 Republic of Korea; 3 Department of Materials Science and Engineering, Korea University, Anam-Dong, Seongbuk-Gu, Seoul, 136-713 Republic of Korea

**Keywords:** Yolk–shell, Nickel oxide, Carbon composite, Anode materials, Spray pyrolysis, Lithium-ion batteries

## Abstract

In this study, coral-like yolk–shell-structured NiO/C composite microspheres (denoted as CYS-NiO/C) were prepared using spray pyrolysis. The unique yolk–shell structure was characterized, and the formation mechanism of the structure was proposed. Both the phase separation of the polyvinylpyrrolidone and polystyrene (PS) colloidal solution and the decomposition of the size-controlled PS nanobeads in the droplet played crucial roles in the formation of the unique coral-like yolk–shell structure. The CYS-NiO/C microspheres delivered a reversible discharge capacity of 991 mAh g^−1^ after 500 cycles at the current density of 1.0 A g^−1^. The discharge capacity of the CYS-NiO/C microspheres after the 1000th cycle at the current density of 2.0 A g^−1^ was 635 mAh g^−1^, and the capacity retention measured from the second cycle was 91%. The final discharge capacities of the CYS-NiO/C microspheres at the current densities of 0.5, 1.5, 3.0, 5.0, 7.0, and 10.0 A g^−1^ were 753, 648, 560, 490, 440, and 389 mAh g^−1^, respectively. The synergetic effect of the coral-like yolk–shell structure with well-defined interconnected mesopores and highly conductive carbon resulted in the excellent Li^+^-ion storage properties of the CYS-NiO/C microspheres.

**Electronic supplementary material:**

The online version of this article (10.1007/s40820-018-0234-0) contains supplementary material, which is available to authorized users.

## Highlights

- Coral-like yolk–shell-structured nickel oxide/carbon composite microspheres were synthesized.
- Phase separation and polystyrene nanobead decomposition affected the structure formation.
- Coral-like yolk with interconnected mesopores provided excellent Li-ion storage properties.

**Electronic supplementary material:**

The online version of this article (10.1007/s40820-018-0234-0) contains supplementary material, which is available to authorized users.

## Graphical abstract

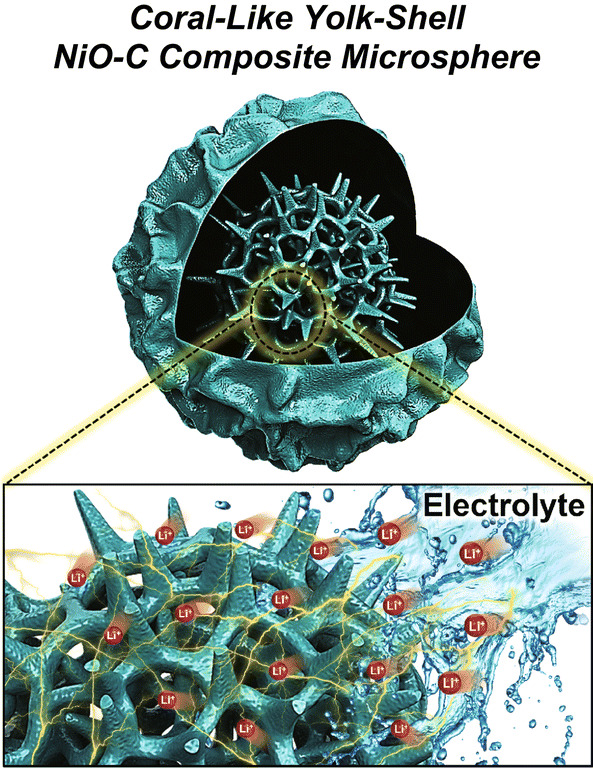

## Introduction

With the rapid increase in the energy demand, lithium-ion batteries (LIBs) have gained immense attention as next-generation energy storage devices and sources of vehicle energy [1–7]. Hence, in order to improve the performance of LIBs, it is imperative to develop innovative anode materials [8–11].Transition metal oxides (TMOs) have been recognized as appropriate anode materials owing to their higher theoretical capacities as compared to that of graphite, high abundance, and chemical stability [12–16]. However, the drastic capacitance fading of TMOs owing to their large volume expansion during cycling has hindered their application as LIB anodes [17–21]. Therefore, various TMO nanostructures including nanoparticles, nanowalls, nanotubes, nanofibers, and nanoflakes have been extensively studied [22–28]. Recently, the yolk–shell structure materials have been used to improve the anode performance of LIBs [29–34]. For example, Zhang et al. synthesized an iron oxide/carbon yolk–shell structure by carbonizing α-Fe_2_O_3_/SiO_2_/poly-dopamine composite nanoparticles followed by the removal of the SiO_2_ layer using NaOH. The Fe_2_O_3_/carbon yolk–shell structure exhibited a high reversible capacity of 810 mAh g^−1^ at 0.2 C rate and an excellent cycling stability while maintaining a capacity of 790 mAh g^−1^ after 100 cycles [32]. Yu et al. also prepared yolk–shell Ni–Co mixed oxide nanoprisms through simple thermal annealing of Ni–Co precursor particles in air. These nanoprisms exhibited a reversible capacity of 1029 mAh g^−1^ after 30 cycles at 200 mA g^−1^ [33]. Furthermore, Kim et al. integrated N-doped carbon in the hollow space between the yolk and the shell to achieve high capacity, accommodate volume change, improve electrical conductivity, and form a stable solid–electrolyte interphase (SEI) layer [34].

However, although yolk–shell structures with various compositions have been studied thus far, their long-term cycle properties are unsatisfactory for practical applications owing to their intrinsic low structural stability. An effective approach to overcome this limitation is to make TMO composites with carbonaceous materials. However, it is difficult to prepare yolk–shell-structured TMO/carbon hybrids using traditional synthesis methods. Therefore, the uniform composition of carbon and TMO and their even distribution in both the yolk and shell are quite challenging and have not been studied before.

In this study, we proposed a novel facile method for the synthesis of yolk–shell-structured TMO/carbon hybrid microspheres. The yolk had a coral-like structure with interconnected mesopores. Coral-like yolks shorten the Li^+^-ion diffusion path, facilitating the penetration of the electrolyte into the yolk during cycling. In addition, yolk–shell carbon composites can expand freely and hence show a highly stable SEI at the surface. Owing to the highly stable SEIs, such composites show excellent Li^+^-ion storage properties. Based on this concept, we synthesized coral-like yolk–shell-structured NiO/C composite microspheres via a one-pot spray pyrolysis process and a subsequent heat treatment. During the spray pyrolysis, polyvinylpyrrolidone (PVP) in the droplet partially phase-separated from the polystyrene (PS) colloidal solution and migrated outward, and interconnected mesopores were formed owing to the decomposition of PS. The subsequent thermal contraction of the inner part of the composite at high reaction temperatures during the spray pyrolysis process resulted in the formation of unique coral-like yolk–shell-structured NiO/C composite microspheres. The resulting NiO/C composite microspheres showed an ideal structure, and their long-term cycling and rate performances were superior to those of the other NiO-based nanomaterials with various morphologies reported till date.

## Experimental Section

### Sample Preparation

Coral-like yolk–shell-structured metal oxide/C composite microspheres were prepared via one-pot spray pyrolysis. First, the NiO–Ni–C composite microspheres with the coral-like yolk–shell structure (denoted as CYS-Ni/NiO/C) were directly prepared by spray pyrolysis using a 0.2 M aqueous spray solution of nickel nitrate hexahydrate [Ni(NO_3_)_2_·6H_2_O, Daejung, 97%], 20 g L^−1^ of PVP [(C_6_H_9_NO), M_w_ 40,000, Daejung], and 20 g L^−1^ of PS nanobeads (40 nm). The size-controlled PS nanobeads (40 nm) were synthesized using an emulsifier-free emulsion polymerization method. The spray pyrolysis system used in this study is shown in Fig. S1. In the spray pyrolysis process, droplets were generated with the aid of a 1.7 MHz ultrasonic spray generator consisting of six vibrators. Subsequently, the droplets were transferred to a quartz reactor (length = 1200 nm and diameter = 50 nm) by N_2_ gas (carrier) at a flow rate of 10 L min^−1^. During the spray pyrolysis process, the reactor temperature was maintained at 700 °C. After the spray pyrolysis process, the as-prepared microspheres (CYS-Ni/NiO/C) were post-treated at 250 °C at a heating rate of 5 °C min^−1^ for 1 h under an air atmosphere in order to optimize the carbon content in the structure and transform the residual metallic Ni into the NiO phase. After the heat treatment, coral-like yolk–shell NiO/C composite microspheres (denoted as CYS-NiO/C) were obtained. For comparison, bare NiO microspheres with a hollow structure (denoted as hollow NiO) were also prepared via spray pyrolysis at 700 °C in an air atmosphere. The spray solution consisted of only nickel nitrate hexahydrate (without PVP and PS nanobeads).

### Characterization Techniques

The morphology of the samples was examined using field-emission scanning electron microscopy (FE-SEM, ULTRA PLUS, ZEISS) and field-emission transmission electron microscopy (FE-TEM, JEOL, JEM-2100F). The phase analysis of the samples was carried out by X-ray diffraction (XRD, D8 Discover with GADDS, Bruker) using Cu K_*α*_ radiation (*λ* = 1.5418 Å). The chemical composition of the samples was investigated by X-ray photoelectron spectroscopy (XPS, Thermo Scientific *K*-Alpha) using a focused monochromatic Al *K*_*α*_ radiation at 12 kV and 20 mA. Raman spectroscopy (Jobin–Yvon LabRam, HR800, excitation source = 514 nm He–Ne laser) was conducted to confirm the presence of a graphitic structure in the samples. The surface areas of the samples were estimated using the Brunauer–Emmett–Teller (BET) method where N_2_ was used as the adsorbate gas. Thermogravimetric analysis (TGA) was carried out using a Pyris 1 TG analyzer (Perkin Elmer) over the temperature range of 25–700 °C at a heating rate of 10 °C min^**−**1^ under an air atmosphere.

### Electrochemical Measurements

The electrochemical performances of the samples as LIB anodes were evaluated using 2032-type coin cells. The prepared NiO samples were used as the working electrode composed of 70 wt% active material, 20 wt% carbon black (Super-P) as the conductive material, and 10 wt% sodium carboxymethyl cellulose as the binder on a copper foil. The Li metal and microporous polypropylene film were used as the counter electrode and separator, respectively. The electrolyte used was 1 M LiPF_6_ in a mixture of fluoroethylene carbonate and dimethyl carbonate with a volume ratio of 1:1. The cells were assembled in a glove box under an Ar atmosphere. The electrochemical performances of the samples were evaluated using cyclic voltammetry (CV), charge–discharge testing, and electrochemical impedance spectroscopy (EIS). The mass loading of the samples for the test was 1.0 mg cm^−2^. The CV measurements of the samples were carried out at a scan rate of 0.1 mV s^−1^ over the potential range of 0.001–3.0 V. The charge–discharge testing of the samples was carried out at current densities of 0.5–10.0 A g^−1^ within the same potential window of 0.001–3.0 V. The EIS of the samples was carried out over the frequency range of 100 kHz–0.01 Hz using a perturbation of 10 mV.

## Results and Discussion

### Synthesis of Ni/NiO/C Microspheres

**Fig. 1:**
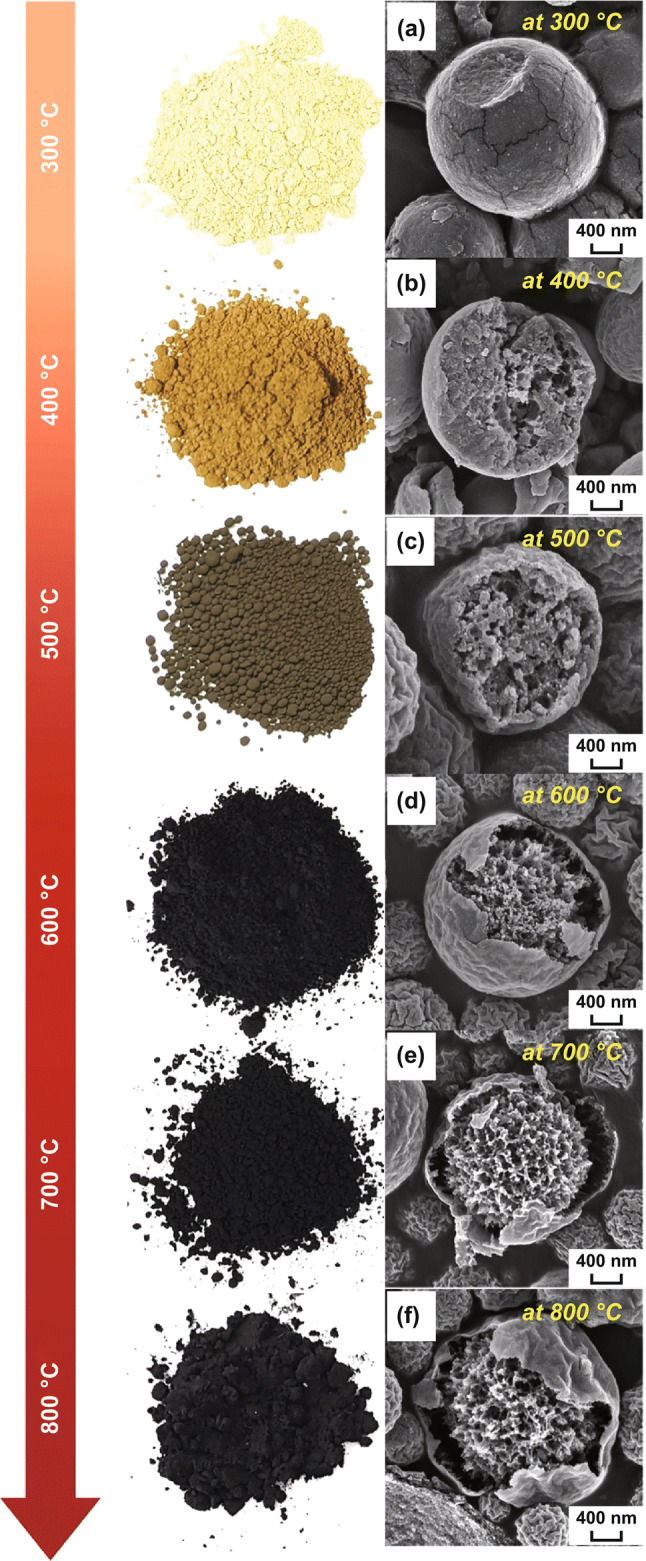
Morphologies of the as-prepared powders obtained by spray pyrolysis at different temperatures: **a** 300 °C, **b** 400 °C, **c** 500 °C, **d** 600 °C, **e** 700 °C, and **f** 800 °C

In order to elucidate the formation mechanism of the unique coral-like yolk–shell-structured metal oxide/C composites, the effects of both the reaction temperature during the spray pyrolysis and organic polymers as additives on the microsphere morphologies were investigated in detail. The morphologies of the as-prepared microspheres obtained using the solution with Ni salt, PVP, and containing an optimum amount of PS nanobeads at various reaction temperatures are shown in Fig. 1. As the reaction temperature was increased from 300 to 700 °C, the decomposition of the Ni salt, PVP, and the PS nanobeads occurred sequentially. It should be noted that the PS nanobeads decomposed at temperatures higher than 500 °C, generating numerous mesopores inside the composite structure (Fig. 1c). Subsequently, the inner part of the composite contracted thermally in the hot reaction zone (at 600 °C) during the spray pyrolysis, which resulted in the formation of a hollow space between the porous yolk and the shell, as shown in Fig. 1d. The formation of the hollow space between the yolk and the shell during the spray pyrolysis process is illustrated in Fig. S2. Therefore, the morphology of the resulting microspheres changed from dense to the desirable coral-like yolk–shell structure. The change in the color of the microspheres from yellow to black indicates the carbonization of PVP. Therefore, coral-like yolk–shell-structured microspheres were obtained at temperatures higher than 600 °C.

**Fig. 2:**
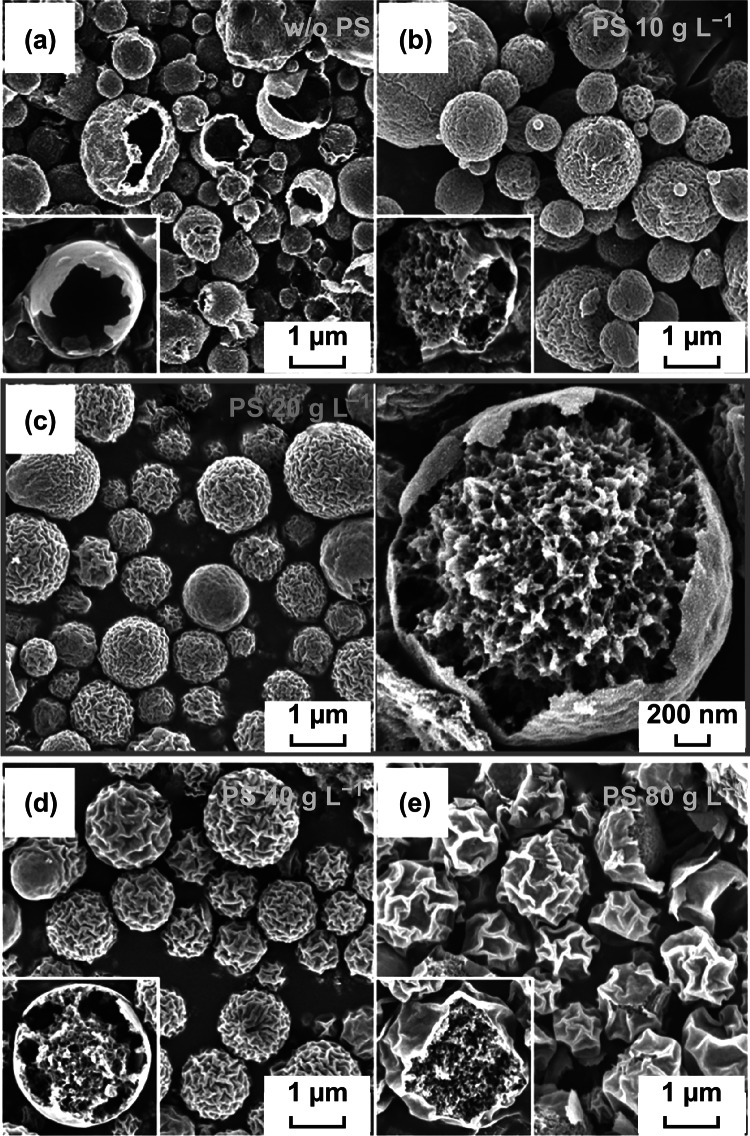
Morphologies of the as-prepared powders obtained from the solution containing Ni salt, PVP, and **a** without PS nanobeads, **b** with 10 g L^−1^, **c** 20 g L^−1^, **d** 40 g L^−1^, and **e** 80 g L^−1^ PS nanobeads

The interaction between the organic polymer additives (PVP and the PS nanobeads) with the Ni salt significantly affected the microsphere morphology (Fig. 2). The microspheres obtained from the Ni salt–PVP solution (without PS nanobeads) were spherical with a hollow structure and thin walls (Fig. 2a). In the spray pyrolysis process, hollow microspheres are formed by the fast drying of the droplets and the rapid decomposition of metal salts at high temperatures. However, when 10 g L^−1^ PS nanobeads were added to the salt solution as an organic additive, fractured core–shell microspheres with a porous core with well-defined mesopores@shell with a wrinkled surface were obtained (Fig. 2b). In the drying step during the initial stage of the spray pyrolysis process, PVP in the droplet partially separated from the PS colloidal solution and migrated outward, while the PS nanobeads in the solution moved inward. At the same time, the Ni salt, which had a high affinity toward PVP, was also transferred outward along with PVP. The decomposition of the PS nanobeads resulted in the generation of mesopores in the structure. As the PS nanobead content of the spray solution was increased to 20 g L^−1^, a hollow space was generated in the region between the porous yolk and the shell, as shown in Fig. 2c. This is because of the decomposition of a large amount of PS nanobeads into gaseous products, which resulted in the generation of numerous mesopores inside the carbon composite structure. Subsequently, the inner part of the composite underwent thermal contraction in the hot reaction zone during the spray pyrolysis process, which resulted in the formation of a hollow space between the yolk and the shell. An increase in the PS nanobead content to 40 g L^−1^ resulted in an increase in the hollow space between the porous yolk and the shell, as shown in Fig. 2d. However, a further increase in the PS nanobead content to 80 g L^−1^ resulted in the shrinking of the shell into the vast internal space formed by excessive PS nanobead decomposition owing to the inward force, which resulted in a core–shell structure with a porous core and shell, as shown in Fig. 2e. In addition, an increase in the PS nanobead content (Fig. 2a–e) resulted in the formation of surface wrinkles because of the migration of PVP to the shell part. Moreover, the depth of the surface wrinkles also increased with an increase in the PS nanobead content owing to the thermal contraction of the shell to the interior of the core. In summary, coral-like yolk–shell-structured metal oxide/C composite microspheres can be prepared by controlling the reaction temperature during the spray pyrolysis process and by using organic additives with an optimum ratio of PVP and PS nanobeads.

**Fig. 3:**
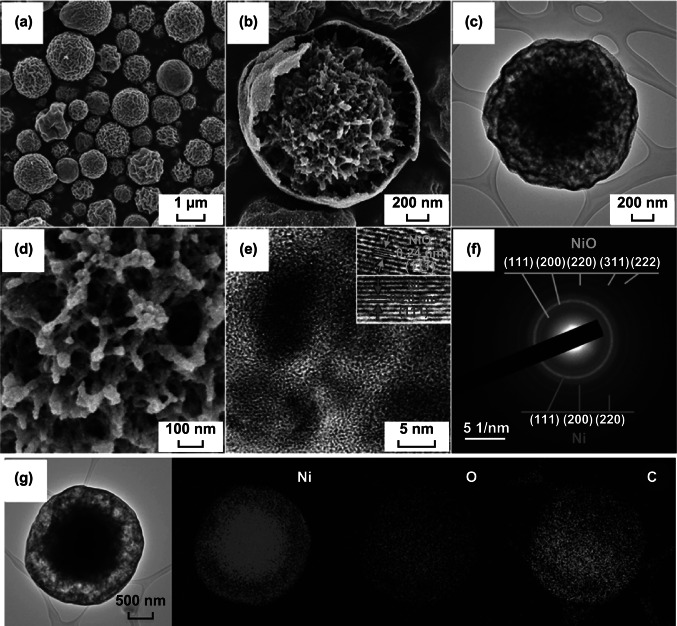
**a**, **b**, **d** FE-SEM images, **c**, **e** HR-TEM images, **f** SAED pattern, and **g** elemental mapping images of the CYS-Ni/NiO/C microspheres

**Fig. 4:**
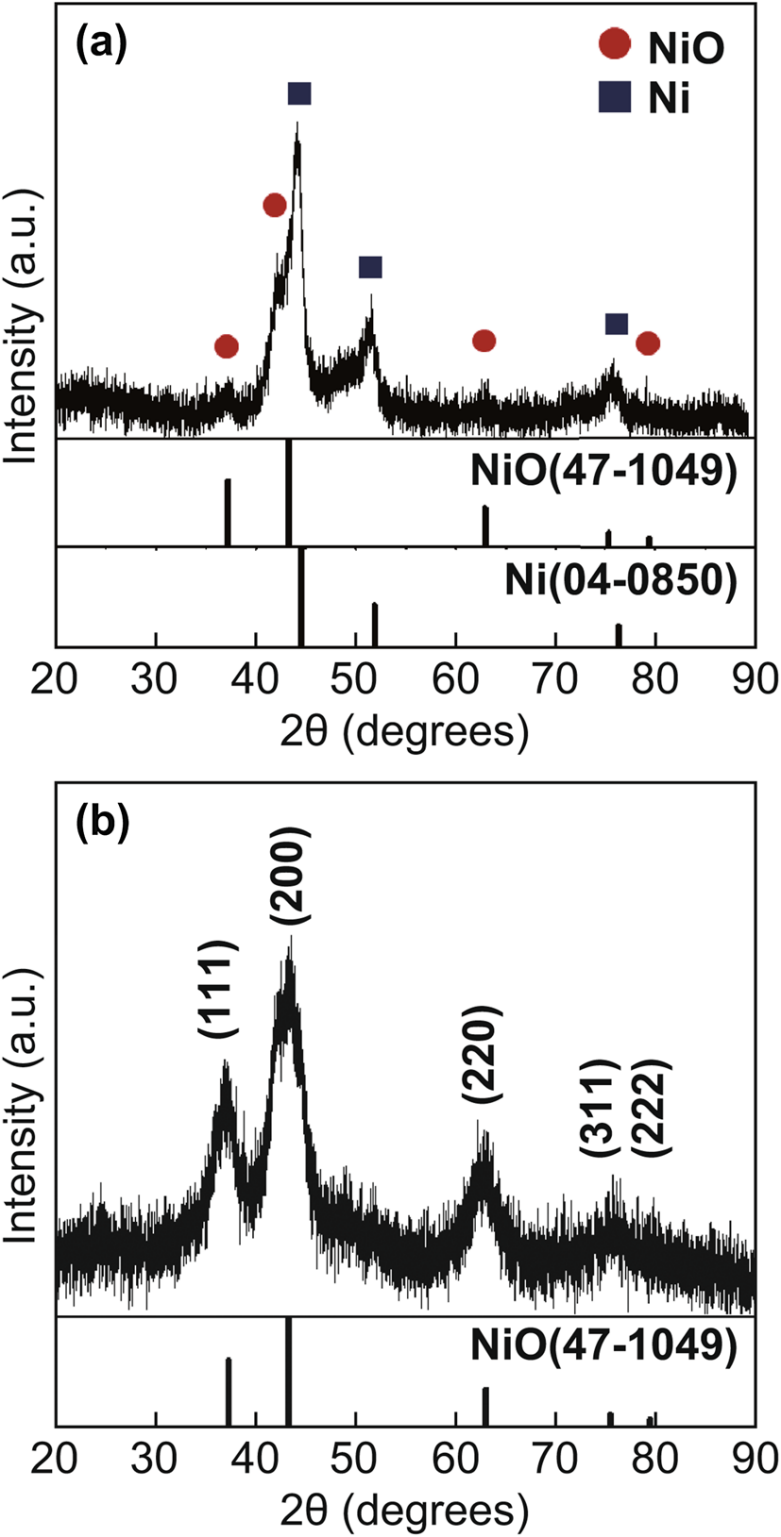
XRD patterns of the **a** CYS-Ni/NiO/C and **b** CYS-NiO/C microspheres

The morphologies of the CYS-Ni/NiO/C composite microspheres prepared by spray pyrolysis at 700 °C using the solution with 20 g L^−1^ of PS nanobeads are shown in Fig. 3. The XRD pattern of these microspheres (Fig. 4a) showed the presence of a predominant metallic Ni phase with cubic NiO phases [35]. This indicates that the microspheres were composed of Ni, NiO, and C (carbonized by PVP). During the spray pyrolysis, a large number of metallic Ni nanocrystals were formed under a N_2_ atmosphere through the carbothermal reduction reaction [36]. The CYS-Ni/NiO/C microspheres were spherical with a mean size of 1.3 µm and showed wrinkled surfaces owing to the shrinkage of the shell at the high reaction temperature of 700 °C. The SEM and TEM images in Fig. 3b, c also showed that the microspheres had a distinct coral-like yolk–void–shell configuration. The microspheres shown in Fig. 3d showed a coral-like yolk with a definite porous structure, and the mesopores were interconnected with each other. The high-resolution TEM image shown in Fig. 3e reveals that the microspheres consisted of nanocrystals (with a size distribution of 5–10 nm) surrounded by a carbon matrix. The Ni and NiO nanocrystals with lattice fringes were separated by gaps of 0.20 and 0.24 nm, which correspond to the (111) plane of the cubic Ni metal and (111) plane of the cubic NiO, respectively [37]. The selected area electron diffraction (SAED) pattern of the composite microspheres (Fig. 3f) also confirmed the presence of metallic Ni and NiO crystals in them. From the elemental mapping images of the microspheres shown in Fig. 3g, it can be observed that Ni was uniformly distributed in the C matrix. The TGA curve shown in Fig. S3a revealed that the microspheres first showed a slight weight gain and then a sharp weight loss. The weight gain at 240 °C can be attributed to the conversion of metallic Ni to NiO. Moreover, the weight loss at 310–400 °C can be attributed to the combustion of C. The weight loss due to the combustion of a large amount of C was reduced by continuous Ni oxidation.

### Synthesis of NiO/C Microspheres

**Fig. 5:**
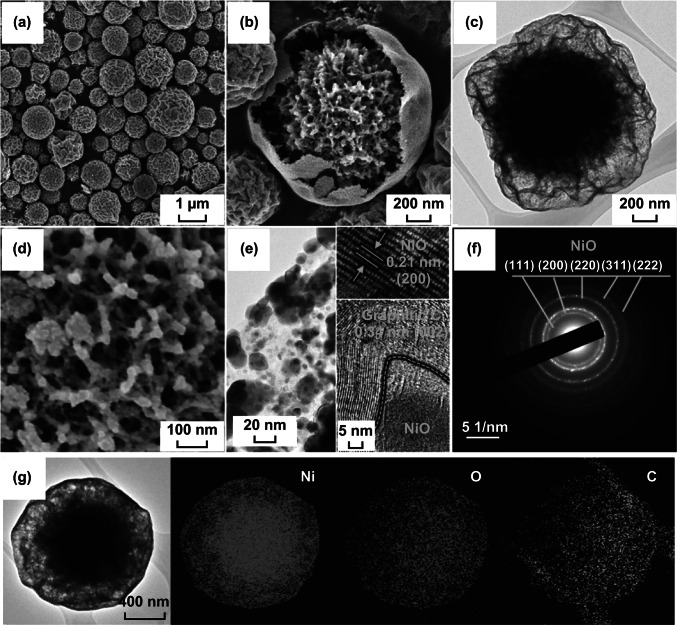
**a**, **b**, **d** FE-SEM images, **c**, **e** HR-TEM images, **f** SAED pattern, and **g** elemental mapping images of the CYS-NiO/C microspheres

In order to optimize C contents and transform metallic Ni into NiO, the CYS-Ni/NiO/C microspheres were heat-treated at 250 °C, and the resulting CYS-NiO/C composite microspheres without metallic Ni are shown in Fig. 5. The XRD pattern of the CYS-Ni/NiO/C microspheres (Fig. 4b) confirmed the complete oxidation of metallic Ni to NiO. The mean crystallite size of NiO was determined by applying the Scherrer equation to its (200) crystal plane and was found to be 17 nm. The CYS-NiO/C microsphere retained its original coral-like yolk–shell structure despite the heat treatment (Fig. 5a–d). In other words, the microspheres showed the coral-like yolk–void–shell, and the mesopores in the yolk were interconnected even after the heat treatment (Fig. 5d). From the high-resolution TEM image shown in Fig. 5e, it can be observed that NiO nanocrystals with a size distribution of 10–20 nm were well distributed in the graphitic carbon matrix. The presence of layers with a lattice spacing of 0.34 nm in the (002) crystal plane confirmed the graphitization of C [38]. Ni metal acted as a catalyst for the graphitization of C during the spray pyrolysis process at a relatively low temperature of 700 °C [39, 40]. The lattice fringes and SAED pattern (inset of Fig. 5e, f) further confirmed the complete conversion of Ni into NiO in the CYS-NiO/C microsphere. The lattice fringes separated by a gap of 0.21 nm corresponded to the (200) crystal plane of cubic NiO [24, 41]. The elemental mapping image of CYS-NiO/C (Fig. 5g) revealed that the NiO nanocrystals were uniformly distributed in the C matrix. The NiO and C contents of the CYS-NiO/C microsphere were 82% and 18%, respectively, as calculated from the TGA results (Fig. S3b).

**Fig. 6:**
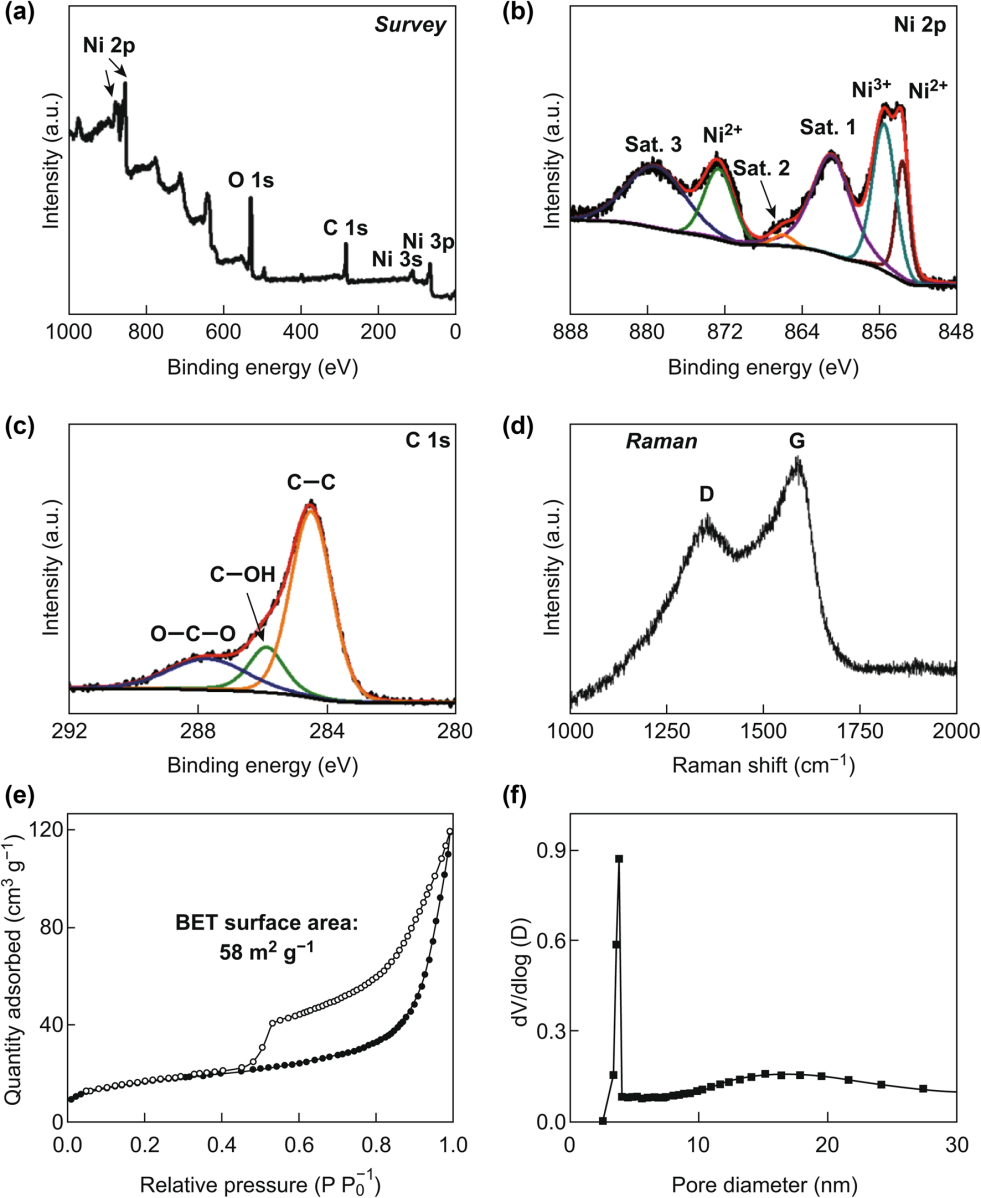
**a** Survey XPS, **b** Ni 2*p* XPS, **c** C 1*s* XPS and **d** Raman spectra, **e** N_2_ adsorption and desorption isotherms, and **f** BJH pore size distribution of the CYS-NiO/C microspheres

The elemental compositions of the CYS-NiO/C microspheres were analyzed using XPS (Fig. 6a–c). The survey spectrum of the CYS-NiO/C microspheres (in Fig. 6a) showed the presence of Ni, O, and C elements, which is consistent with the elemental mapping results (Fig. 5g) [42]. The high-resolution Ni 2*p* spectrum of the microspheres (Fig. 6b) showed two major Ni 2*p*_3/2_ and Ni 2*p*_1/2_ peaks for Ni^3+^ and Ni^2+^, respectively, along with their satellite peaks [43, 44]. The Ni^3+^ peak can be attributed to the formation of O-rich nickel oxide owing to the burning of C during the heat treatment [43, 44]. However, the amount of O-rich nickel oxide was assumed to be negligible because of the existence of a pure NiO phase, as confirmed by the XRD results (Fig. 4b). Figure 6c shows the deconvoluted C1*s* spectrum of the microspheres. The peaks corresponding to *sp*^2^-bonded C (C–C), C–OH, and O–C–O were observed at 284.5, 285.9, and 287.7 eV, respectively [45]. The sharp peak corresponding to *sp*^2^-bonded C observed in the XPS and Raman spectra of the CYS-NiO/C microspheres (Fig. 6d) further confirmed the presence of graphitic carbon in them. The presence of graphitic carbon can be attributed to the graphitization of C, wherein the Ni metal acted as a catalyst. This is consistent with the high-resolution TEM results (Fig. 5e). The *I*_D_*/I*_G_ ratio (measure of degree of graphitization) of the CYS-NiO/C microspheres was approximately 0.72. The N_2_ adsorption and desorption isotherms (Fig. 6e) and pore distribution curves (Fig. 6f) of the CYS-NiO/C microspheres revealed their porous nature. The isotherms of the CYS-NiO/C microspheres were H3 type, indicating the presence of mesopores within the structure [46]. The BET surface area of the CYS-NiO/C microspheres was 58 m^2^ g^−1^. The CYS-NiO/C microspheres showed two types of mesopores, wherein the mesopores smaller than 10 nm can be attributed to the spaces uniformly distributed in the NiO/C composite, and the relatively larger mesopores can be attributed to the voids formed by the decomposition of PS nanobeads. Additionally, a very sharp peak at 3.8 nm was observed because of the tensile strength effect evident from the pore distribution curve [47, 48]. The numerous well-developed mesopores in the structure and C matrix contributed to the high BET surface area of the CYS-NiO/C microspheres.

**Scheme 1:**
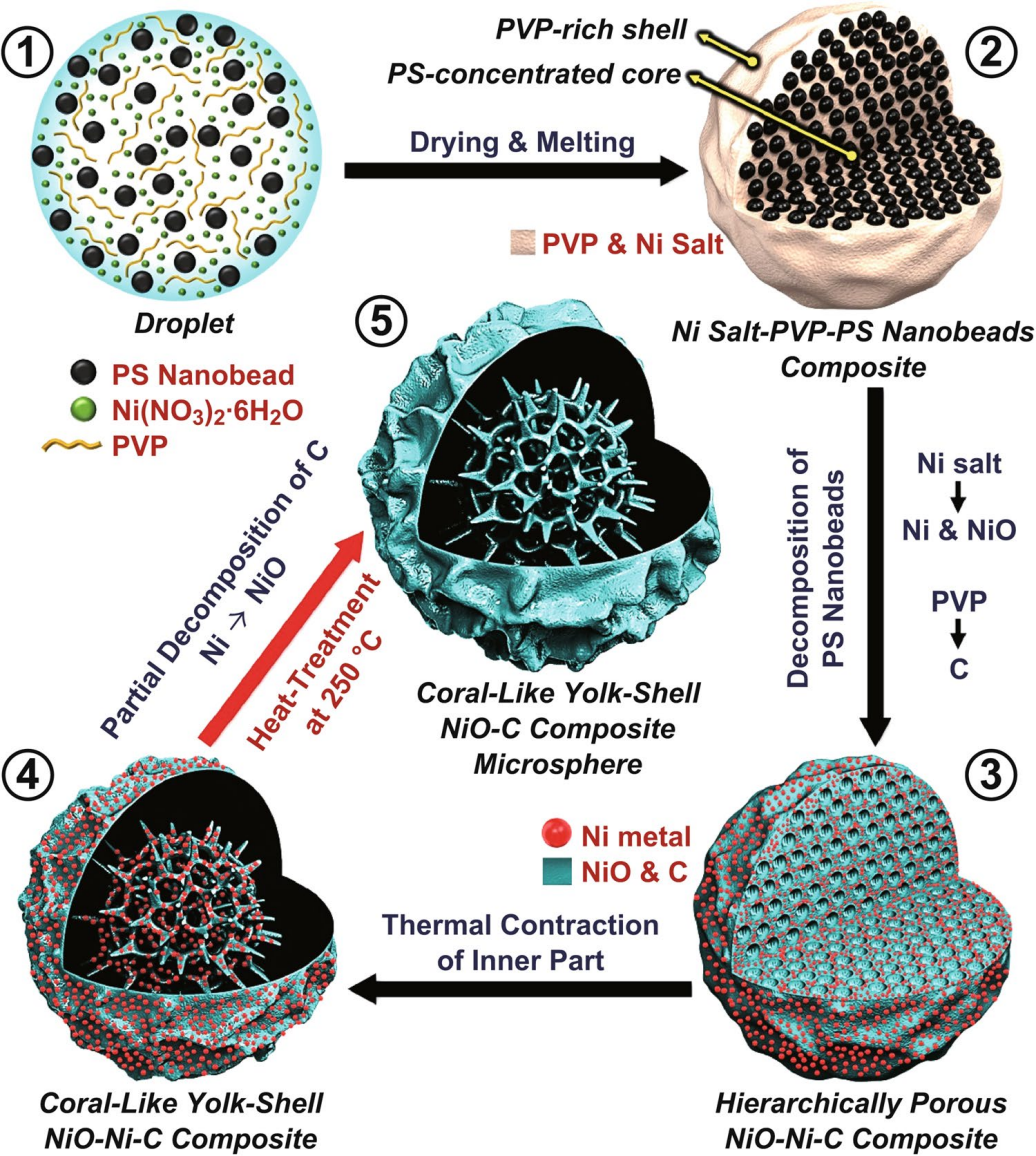
Formation mechanism of the coral-like yolk–shell-structured NiO/C composite microspheres

The formation mechanism of the CYS-NiO/C composite microspheres can be elucidated as follows: Droplets consisting of uniformly distributed Ni salt, PVP, and PS nanobeads were generated by an ultrasonic nebulizer during the spray pyrolysis process (Scheme 1-①). PVP bonded with PS in water through the interaction between the hydrophobic PS methylene/methane groups and the positive dipole moment of the pyrrolidone ring amide nitrogen. Additionally, the amide groups of PVP bonded with Ni ions through strong ionic bonds. Therefore, PVP stabilized the PS nanobeads and Ni salt in the droplets generated during the spray pyrolysis process. Upon drying, each droplet (several microns in size) produced one Ni salt–PVP composite microsphere containing numerous 40-nm PS nanobeads. During the spray pyrolysis, PVP melted and partially phase-separated from the PS colloidal solution. It then migrated to the outside of the structure. At the same time, the Ni salt, which had a high affinity toward PVP, also moved outside the structure along with PVP. Here, PS nanobeads were homogeneously dispersed throughout the microspheres except the PVP-rich shell. Hence, a Ni salt–PVP–PS nanobead composite with a PVP-rich shell and PS-concentrated core was formed (Scheme 1-②). Subsequently, several changes occurred simultaneously, resulting in the formation of a hierarchically porous NiO–Ni–C composite: (1) the decomposition of nickel salt to metallic Ni and NiO nanocrystals, (2) carbonization of PVP, and (3) formation of mesopores because of the decomposition of PS nanobeads (Scheme 1-③). The subsequent thermal contraction of the inner part of the structure resulted in the generation of a hollow space between the porous yolk with well-defined voids and the shell in the CYS-Ni/NiO/C microsphere (Scheme 1-④). Finally, the oxidation of the metallic Ni and partial decomposition of the C matrix in the CYS-Ni/NiO/C microsphere during the post-heat treatment resulted in the formation of a CYS-NiO/C composite microsphere (Scheme 1-⑤).

### Evaluation of Li-Ion Storage Performance

**Fig. 7:**
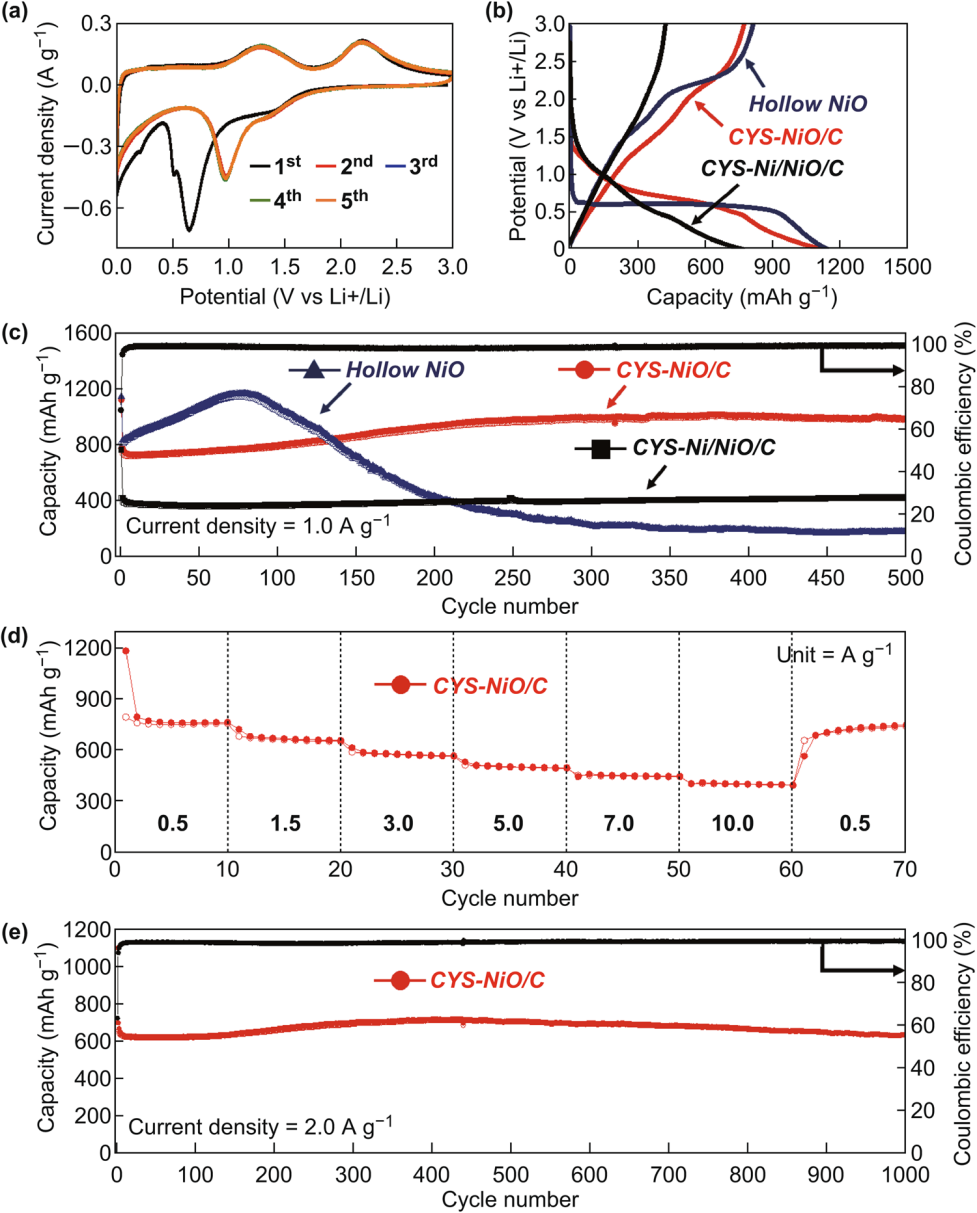
Electrochemical properties of the CYS-NiO/C, CYS-Ni/NiO/C, and hollow NiO microspheres for lithium-ion storage: **a** CV curves of the CYS-NiO/C microspheres, **b** 1st charge/discharge curves at the current density of 1.0 A g^−1^, **c** cycling performance at the current density of 1.0 A g^−1^, **d** rate performance of the CYS-NiO/C microspheres, and **e** long-term cycling performance of the CYS-NiO/C microspheres at the current density of 2.0 A g^−1^

The effects of the morphological features of the CYS-NiO/C and CYS-Ni/NiO/C microspheres on their electrochemical performance as LIB anodes were investigated. Hollow-structured bare NiO microspheres were directly prepared by spray pyrolysis using a nickel nitrate solution (without both PVP and PS nanobeads) under an air atmosphere for comparison, as shown in Fig. S4. The surface of the droplet was supersaturated by fast drying, which precipitated NiO crystals on the shell. Subsequently, the Ni salts in the droplet diffused out, resulting in the formation of a NiO microsphere with a hollow structure. The CV curves for the first five cycles of the CYS-NiO/C microspheres at a scan rate of 0.1 mV s^−1^ over the potential range of 0.001–3.0 V (versus Li^+^/Li) are shown in Fig. 7a. In the first cathodic scan, a reduction peak was observed at approximately 0.64 V attributing to the initial reduction of NiO to Ni accompanied by the formation of amorphous Li_2_O and the decomposition of the electrolyte to form an SEI [24, 49–51]. The relatively weak peak at 0.51 V can be attributed to structural destruction and was not observed in the subsequent scans [49]. Li^+^-ion insertion into the graphitic C matrix was also observed at 0.21 V [50]. The reduction peaks shifted to higher potentials from the second cycle onwards because of the formation of ultrafine NiO nanocrystals during cycling [24, 51]. In the anodic scan, from the first cycle onwards, two broad peaks were observed at 1.29 and 2.18 V corresponding to the dissolution of the organic SEI layer and the subsequent oxidation of the Ni nanocrystals into NiO along with the decomposition of Li_2_O [24, 49–51]. The excellent reversibility of the discharge–charge process described by the reaction: ${\text{NiO }} + 2{\text{Li }}^{ + } + 2{\text{e}}^{ - } \leftrightarrow {\text{Ni}} + {\text{Li}}_{ 2} {\text{O}}$, was ensured by the overlapped CV profiles in Fig. 7a after the second cycle [50, 51]. The CV curves of the CYS-Ni/NiO/C and hollow NiO microspheres are shown in Fig. S5. The CYS-Ni/NiO/C microspheres showed relatively broad CV peaks with low intensities as compared to the hollow NiO microspheres because of their low-crystalline NiO composition and high Ni content. An extra reduction peak was observed at 1.1 V in the first cathodic scan because of the decomposition of the electrolyte and the formation of SEI films [52]. The CV curve of the hollow NiO microsphere exhibited a sharp reduction peak at approximately 0.48 V owing to the formation of amorphous Li_2_O and the SEI and the reduction of NiO into Ni [24, 49–51]. Among the samples, the CYS-NiO/C microspheres showed a high-potential reduction peak in the first cathodic scan, indicating that their Li^+^-ion lithiation/delithiation reactions proceeded readily.

The initial discharge–charge profiles of the CYS-NiO/C, CYS-Ni/NiO/C, and hollow NiO microspheres at a high current density of 1.0 A g^−1^ are shown in Fig. 7b. The discharge–charge profiles of the samples were consistent with their CV results. The discharge curve of the hollow NiO microspheres featured a clear long plateau at approximately 0.59 V owing to the presence of highly crystalline NiO crystals in them [24, 51]. However, the CYS-NiO/C and CYS-Ni/NiO/C microspheres exhibited an unclear plateau because of the presence of C-surrounded NiO crystals (low crystallinity) in them [24, 53]. The initial discharge capacities of the CYS-NiO/C, CYS-Ni/NiO/C, and hollow NiO microspheres were 1124, 770, and 1148 mAh g^−1^, respectively, and their corresponding charge capacities were 778, 426, and 819 mAh g^−1^, respectively. The theoretical capacity of the CYS-NiO/C microspheres was about 588 mAh g^−1^, as calculated using the theoretical specific capacities of NiO (718 mAh g^−1^) and C (372 mAh g^−1^). The high capacity of the CYS-NiO/C microspheres can be attributed to the partial reversible formation and decomposition of the gel-like SEI film on the surface of the electrode and their pseudo-capacitance [54].The initial Coulombic efficiencies (CE) of the CYS-NiO/C, CYS-Ni/NiO/C, and hollow NiO microspheres were found to be 69%, 55%, and 71%, respectively. The CYS-Ni/NiO/C microspheres showed the lowest CE among the samples because of their high C content with a high initial irreversible capacity loss [24, 30]. Although the CYS-NiO/C microspheres also had a C content of 18 wt%, their CE was comparable to that of the C-free NiO hollow microspheres. The high structural stability of the CYS-NiO/C microspheres in the first discharge and charge cycles resulted in a high initial CE.

The cycling performances of the samples at the current density of 1.0 A g^−1^ are shown in Fig. 7c. The hollow NiO microspheres showed a gradual increase in the capacity up to 80 cycles. The initial increase in the capacity was due to their pulverization with large NiO crystals, which resulted in the generation of a fresh metal surface in every cathodic process and the formation of a continuous reversible SEI layer [46, 54, 55]. However, the microspheres showed a drastic decrease in the capacity to 312 mAh g^−1^ after 250 cycles because of the collapse of their structure by large volume changes during the repeated cycles. In contrast, both the CYS-NiO/C and CYS-Ni/NiO/C microspheres exhibited excellent cycling performances even at the high current density of 1.0 A g^−1^. The CYS-NiO/C microspheres showed a higher specific capacity than that of the CYS-Ni/NiO/C microspheres. This is because the CYS-Ni/NiO/C microspheres are composed of metallic Ni with inactivity for the LIB reaction and a relatively larger amount of C content with a low discharge capacity in the structure [52, 53, 56]. The CYS-NiO/C, CYS-Ni/NiO/C, and hollow NiO microspheres delivered reversible specific discharge capacities of 991, 430, and 191 mAh g^−1^ after 500 cycles, respectively. The CYS-NiO/C microspheres maintained a steady CE of more than 99.3%. Because of the C-surrounded NiO crystals, interconnected mesopores in the core, and hollow space between the yolk and the shell, the CYS-NiO/C microspheres effectively accommodated the volume expansion induced by the repeated lithiation/delithiation of Li^+^ ions and showed high cycling stability. Moreover, the fast Li^+^ ion and electron diffusion in these microspheres resulted in a superior rate performance, as shown in Fig. 7d. The final discharge capacities of the CYS-NiO/C microspheres at the current densities of 0.5, 1.5, 3.0, 5.0, 7.0, and 10.0 A g^−1^ were 753, 648, 560, 490, 440, and 389 mAh g^−1^, respectively. The coral-like yolk with numerous interconnected mesopores provided easy electrolyte accessibility to the electrode, thus providing a short diffusion length for Li^+^ ions, which resulted in an excellent rate performance. When the current density was reduced to 0.5 A g^−1^ again, the discharge capacity of the CYS-NiO/C microspheres recovered well to 737 mAh g^−1^, indicating that their Li^+^-ion storage performance was not degraded even at high current densities. Since the capacity of the hollow NiO microspheres increased gradually up to 80 cycles (Fig. S6), they showed a higher capacity than the CYS-NiO/C microspheres at the same current density. This can be attributed to the pulverization of the hollow NiO microspheres with large NiO crystals, which resulted in the generation of a fresh metal surface in every cathodic process and the formation of a reversible SEI layer continuously up to 80 cycles.

The long-term cycling performance and CE of the CYS-NiO/C microspheres at the high current density of 2.0 A g^−1^ are shown in Fig. 7e. The discharge capacities at the 2nd and 1000th cycles were 699 and 635 mAh g^−1^, respectively, and the capacity retention calculated from the second cycle was 91%. The CE of the CYS-NiO/C microspheres reached 99.1% after the 15th cycle and remained constant during the subsequent cycles. The CYS-NiO/C microspheres showed the best reversible capacities at high current densities and long-term cycling performance as compared to the other NiO materials and their carbon hybrids with various morphologies reported previously (Table S1). This can be attributed to the synergetic effect of the coral-like yolk–shell structure with well-defined interconnected mesopores and conductive carbon in these microspheres.

**Fig. 8:**
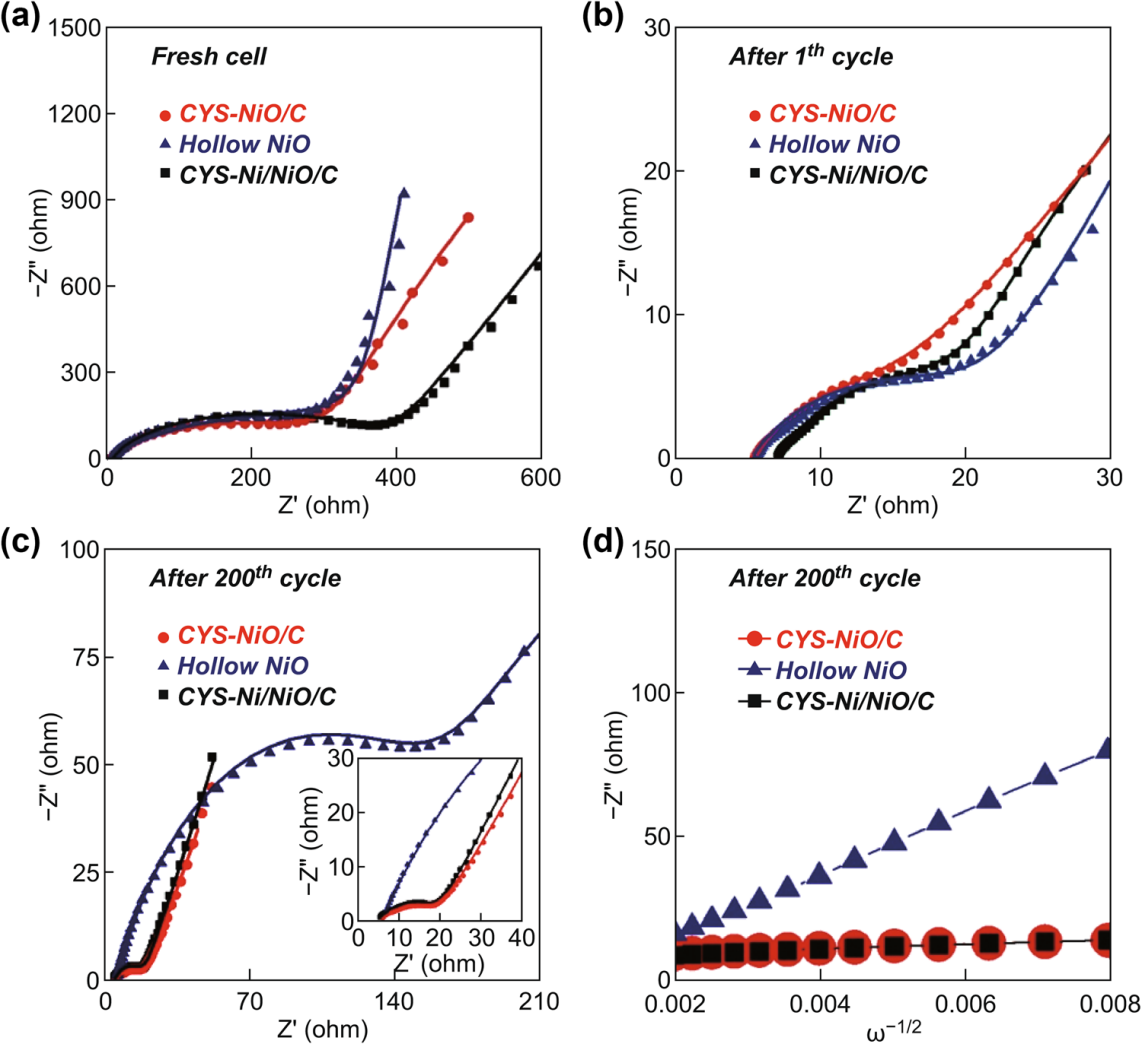
**a**–**c** Nyquist impedance plots (lines represent fitting data) and **d** relationships between the real part of the impedance (*Z*_re_) and *ω*^−1/2^ of the CYS-NiO/C, CYS-Ni/NiO/C, and hollow NiO microspheres: **a** before cycling, **b** after the 1st cycle, **c, d** after the 200th cycle

**Fig. 9:**
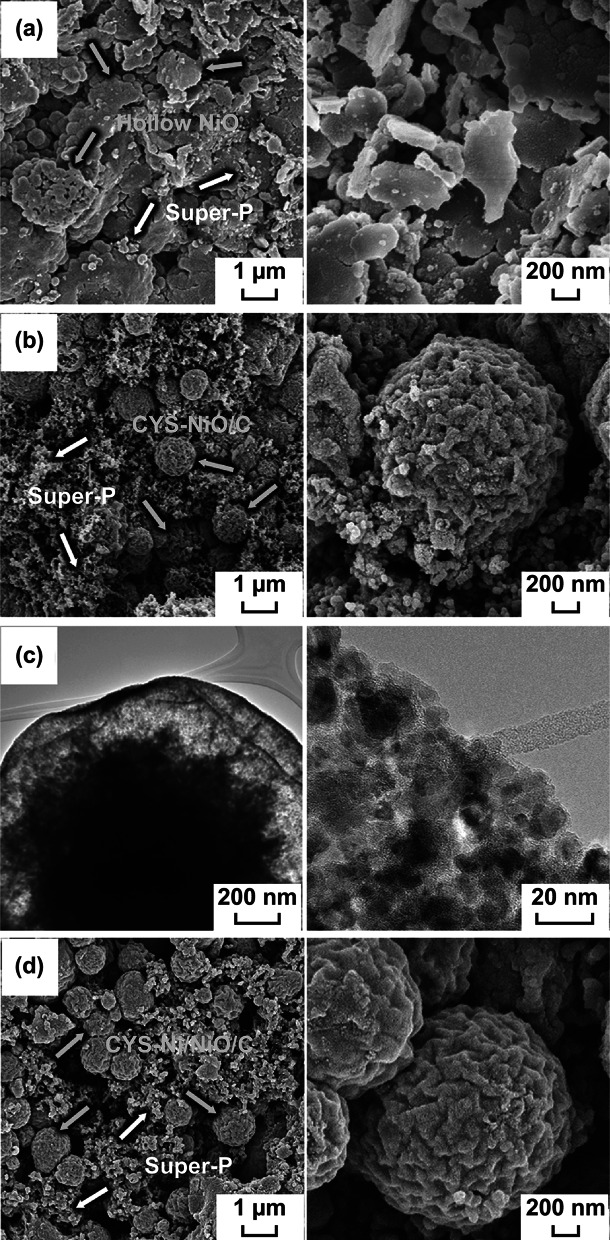
Morphologies of **a** hollow NiO, **b**, **c** CYS-NiO/C, and **d** CYS-Ni/NiO/C microspheres after the 200th cycles: **a**, **b**, **d** FE-SEM images and **c** TEM images

EIS was carried out to investigate the Li^+^-ion storage kinetics of the CYS-NiO/C, CYS-Ni/NiO/C, and hollow NiO microspheres. The Nyquist plots of the samples before cycling and after the 200th cycle were obtained via deconvolution using a Randle-type equivalent circuit model, as shown in Fig. S7. The medium-frequency semicircles in the Nyquist plots correspond to the charge-transfer resistance (*R*_ct_) between the active material and the electrolyte, while the low-frequency region corresponds to the diffusion of Li^+^ ions within the electrodes [57–59]. The *R*_ct_ values of the CYS-NiO/C, CYS-Ni/NiO/C, and hollow NiO microspheres before cycling were 314, 418, and 374 Ω, respectively, as shown in Fig. 8a. Although the CYS-Ni/NiO/C microspheres consisted of a large amount of C, the presence of amorphous C contributed to the highest *R*_ct_ value. However, the *R*_ct_ values of the microspheres decreased abruptly after the 1st cycle because of the formation of ultrafine NiO nanocrystals, as shown in Fig. 8b. The *R*_ct_ values of the CYS-NiO/C, CYS-Ni/NiO/C, and hollow NiO microspheres after the 1st cycle were 15, 22, and 25 Ω, respectively. After 200 cycles, both the CYS-NiO/C and CYS-Ni/NiO/C microspheres with the coral-like yolk–shell structure showed low *R*_ct_ values of 18 and 19 Ω, respectively. This suggests that these microspheres showed high structural stability during the repeated Li^+^-ion lithiation/delithiation processes, as shown in Fig. 8c. The presence of C-surrounded NiO crystals, interconnected mesopores in the core, and hollow space between the yolk and shell improved the structural stability of the samples. In addition, the easy electrolyte accessibility of the electrode and the short diffusion length of Li^+^ ions lowered the charge-transfer resistance of the microspheres. However, the structural destruction of the hollow NiO microspheres during cycling significantly increased their *R*_ct_ value to 223 Ω. Figure 8d shows the relationship between the *Z*_re_ and *ω*^−1/2^ [of the samples *ω* is the angular frequency in the low-frequency region (*ω* = 2*πf*)] in the low-frequency region after 200 cycles. The low slope of the fitted curve of the electrodes at low frequencies indicates their good Li^+^-ion kinetics in the electrode materials. Both the CYS-NiO/C and CYS-Ni/NiO/C microspheres showed lower gradients than that of the hollow NiO microspheres. This result suggests that the CYS-NiO/C and CYS-Ni/NiO/C microspheres showed better diffusion of the Li^+^-ion diffusion than the hollow NiO microspheres. The interconnected mesopores in the core and the hollow space between the yolk and shell of the coral-like yolk–shell structures allowed the easy electrolyte accessibility to the electrode and shortened the diffusion length of Li^+^ ions. In order to confirm this, the morphologies of the samples were examined after 200 cycles. Figure 9a clearly shows the cycle-induced structural collapse of the hollow NiO microspheres. However, the CYS-NiO/C and CYS-Ni/NiO/C microspheres showed excellent structural stability for repeated Li^+^-ion insertion and desertion over 200 cycles (Fig. 9b and d, respectively). The CYS-NiO/C microspheres maintained their coral-like yolk–shell structure (with the shell composed of NiO and C) (TEM image shown in Fig. 9c). The unique microspheres exhibited long-term cycling and high rate performances during repeated Li^+^-ion insertion and desertion because of the synergetic effect of the coral-like yolk–shell structure with well-defined interconnected mesopores and conductive carbon.

## Conclusions

In this study, coral-like yolk–shell-structured metal oxide/carbon composite microspheres were prepared using spray pyrolysis for the first time. During the spray pyrolysis, PVP in the droplet partially phase-separated from the PS colloidal solution and migrated outward, and interconnected mesopores were formed by the decomposition of PS. The subsequent thermal contraction of the inner part of the composites at high reaction temperatures during the spray pyrolysis resulted in the formation of unique CYS-NiO/C microspheres. The CYS-NiO/C microspheres exhibited excellent electrochemical properties for Li^+^-ion storage because of their high structural stability, shortened Li^+^-ion diffusion paths, high electrical conductivity, and easy penetration of the electrolyte into the yolk during the repeated Li^+^ lithiation/delithiation processes. We believe that this novel strategy can be used for designing and synthesizing unique coral-like yolk–shell-structured metal oxide/carbon composites for a wide range of applications such as catalysis, gas sensors, and hydrogen evolution reactions, and energy storage.

## Electronic Supplementary Material

Below is the link to the electronic supplementary material.

Supplementary material 1 (PDF 1103 kb)
