# Supplementary material for: Coral-Like Yolk–Shell-Structured Nickel Oxide/Carbon Composite Microspheres for High-Performance Li-Ion Storage Anodes
Source: Nanomicro Lett. 2019 Jan 9;11:3. doi: 10.1007/s40820-018-0234-0 (PMC7770980; doi:10.1007/s40820-018-0234-0)
Supplement: Supplementary file 1 — Supplementary material 1 (PDF 1103 kb) [file 40820_2018_234_MOESM1_ESM.pdf]

Supporting Information for

Coral-Like Yolk-Shell-Structured Nickel Oxide/Carbon Composite Microspheres for High-Performance Li-Ion Storage Anodes

Min Su Jo[1], Subrata Ghosh[2], Sang Mun Jeong[2], Yun Chan Kang[3,*], Jung Sang Cho[1,*]

[1]Department of Engineering Chemistry, Chungbuk National University, Chungbuk 361-763, Republic of Korea

[2]Department of Chemical Engineering, Chungbuk National University, Chungbuk 361-763, Republic of Korea

[3]Department of Materials Science and Engineering, Korea University, Anam-Dong, Seongbuk-Gu, Seoul 136-713, Republic of Korea

*Corresponding authors. E-mail: jscho@cbnu.ac.kr (Jung Sang Cho), yckang@korea.ac.kr (Yun Chan Kang)

Supplementary Figures

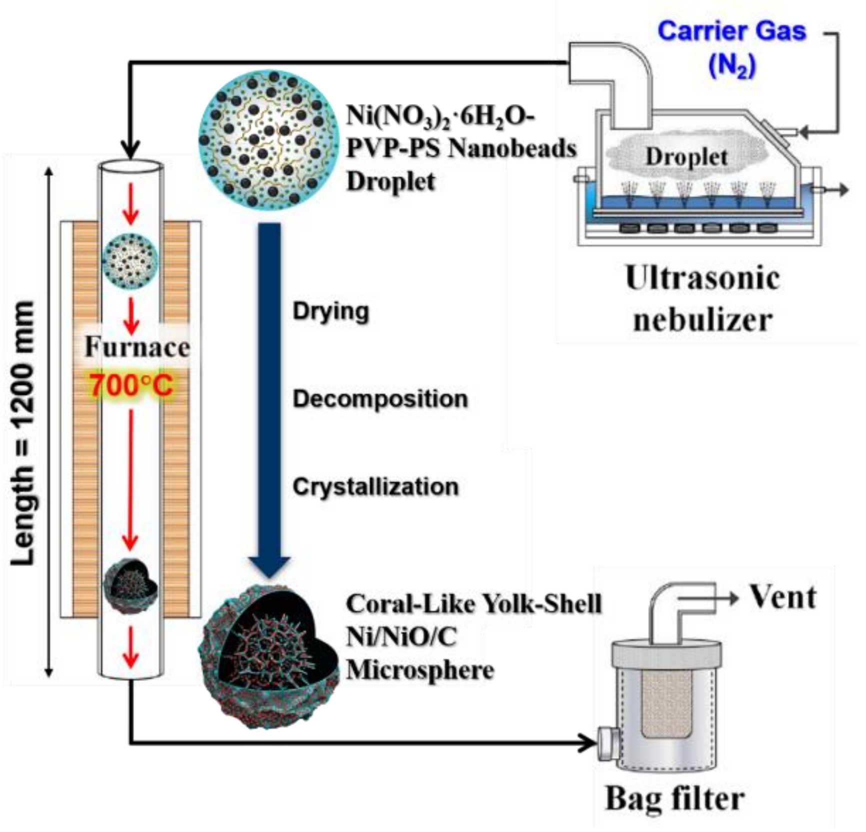

Fig. S1 Schematic diagram of the spray pyrolysis system for the synthesis of CYS-Ni/NiO/C microspheres

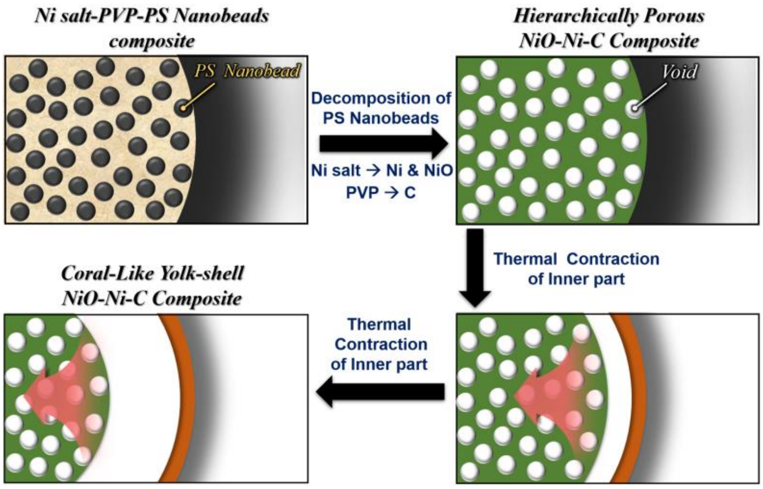

Fig. S2 Detail formation mechanism of the hollow space between yolk and shell during spray pyrolysis

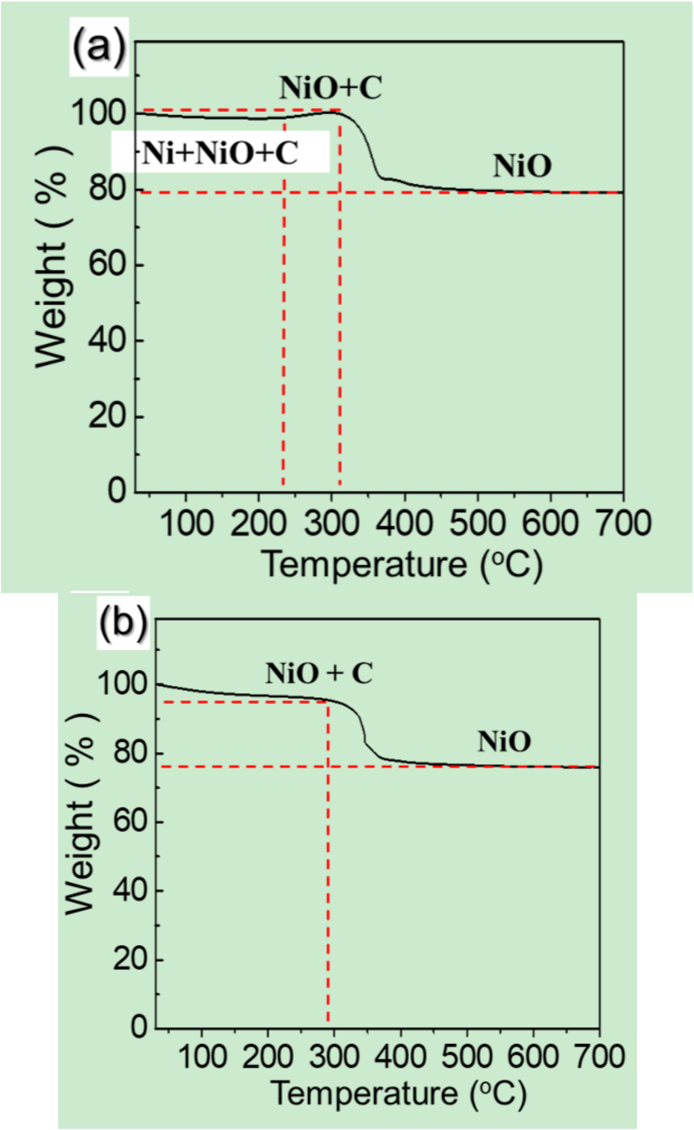

Fig. S3 TG curve of **a** CYS-Ni/NiO/C microsphere, and **b** CYS-NiO/C microsphere

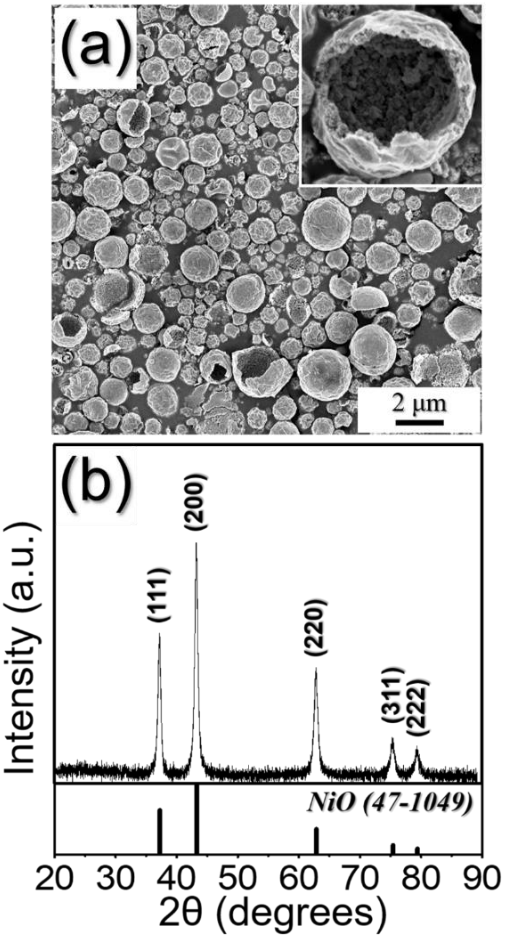

Fig. S4 a SEM images, and **b** XRD pattern of the hollow NiO microspheres

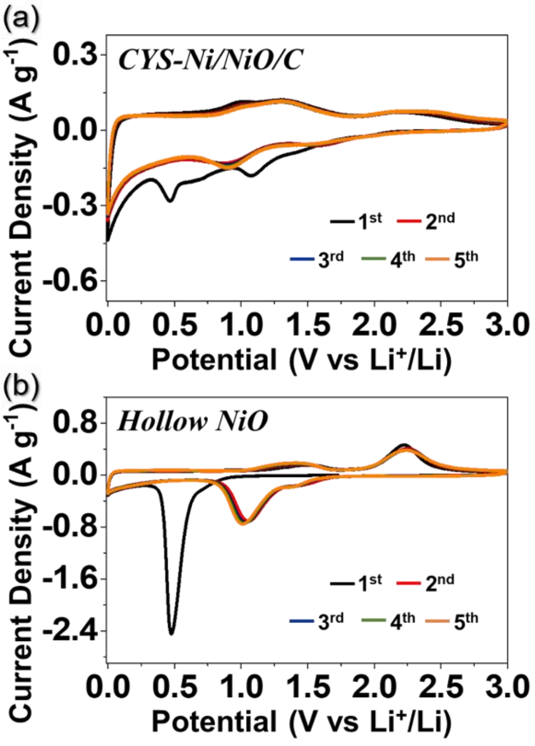

Fig. S5 Cyclic voltammogram (CV) curves of **a** CYS-Ni/NiO/C microspheres and **b** hollow NiO microspheres

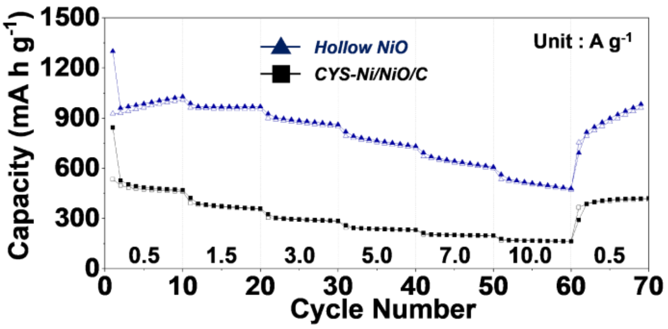

Fig. S6 Rate performances of CYS-Ni/NiO/C and hollow NiO microspheres at different current densities

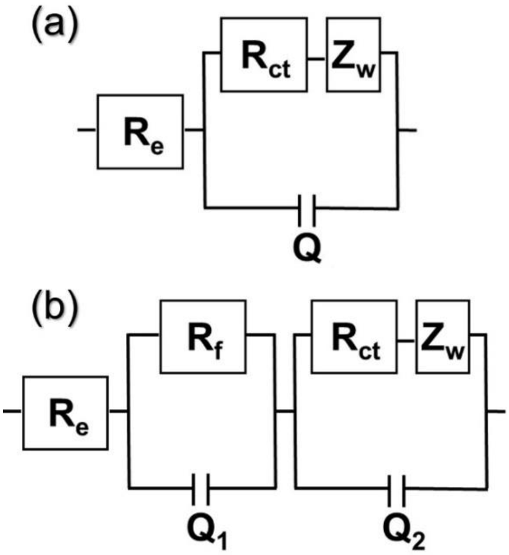

Fig. S7 Equivalent circuit model used for AC impedance fitting: **a** bfore cycling, and **b** after cycling, R_{ct} = charge-transfer resistance, R_e = electrolyte resistance, R_f = SEI layer resistance, Q_1 = dielectric relaxation capacitance, Q_2 = associated double layer capacitance

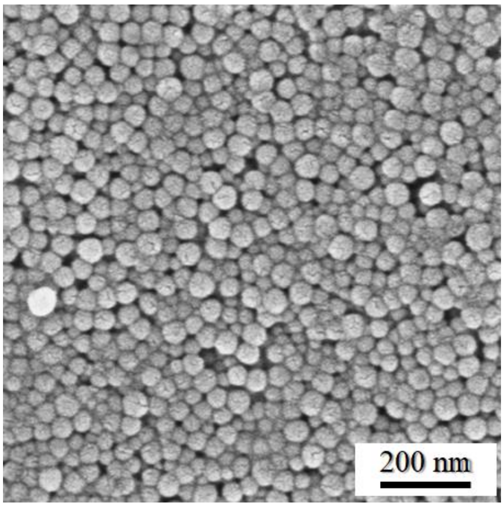

Fig. S8 SEM image of the PS nanobeads with 40 nm synthesized using an emulsifier-free emulsion polymerization method

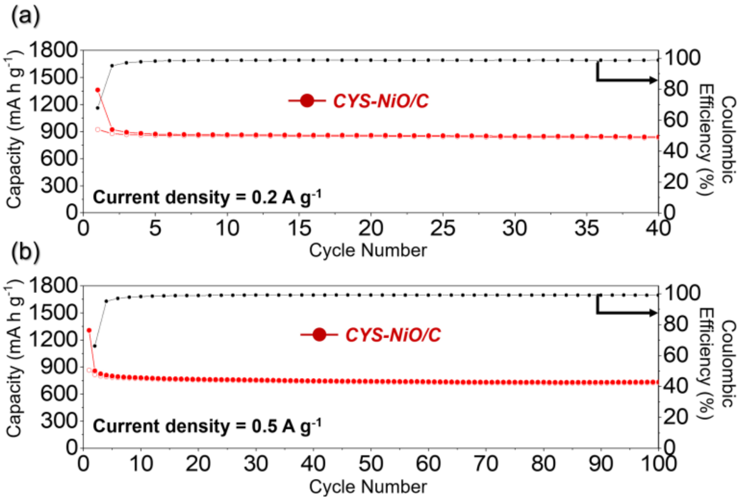

Fig. S9 Cycle properties of CYS-NiO/C microsphere at current densities of **a** 0.2 A g^{-1} and **b** 0.5 A g^{-1}

The authors investigated the contribution of carbon to the capacities of CYS-NiO/C microspheres for Li^+ ion storage. In order to calculate the capacity contribution of the C, CYS-NiO/C microspheres was etched with HCl solution to obtain pure C powders. From the EDS and CV measurement in Fig. S10b, c, the complete removal of NiO in the structure was confirmed. The first discharge–charge profile confirms the powders to be pure C with discharge/charge capacities of 1137 and 568 mAh g^{-1}, respectively as shown in Fig. S10d. The pure C powders exhibited a reversible discharge capacity of 339 mAh g^{-1} at a current density of 1.0 A g^{-1} for the 200th cycle as shown in Fig. S10e. Therefore, the contribution of the C to the discharge capacity of CYS-NiO/C microspheres could be estimated to be 6.2%.

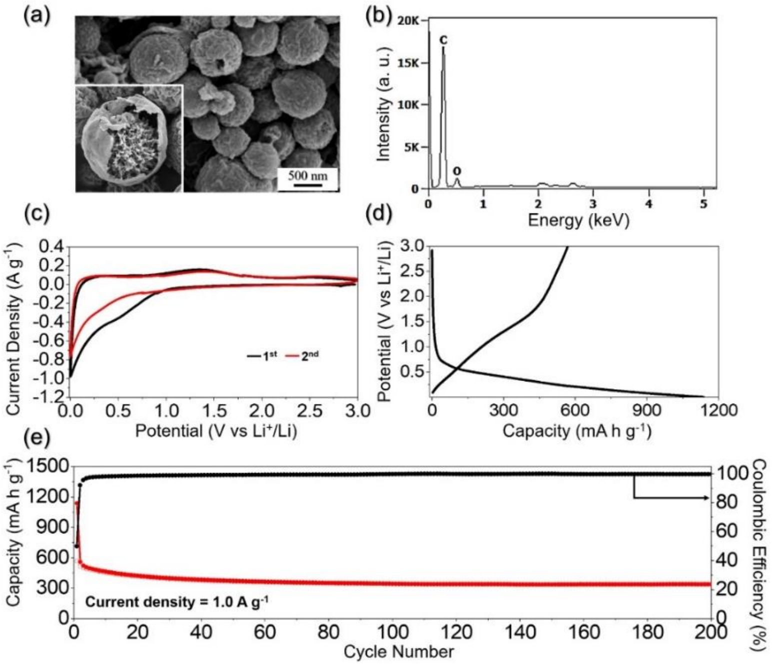

Fig. S10 a FE-SEM image, **b** EDS spectrum, **c** CV curve, **d** Initial discharge/charge curves, and **e** cycling performance of C powders prepared from CYS-NiO/C microspheres by etching with HCl solution

The authors obtained powders from a solution containing Ni salt, PVP, and without PS nanobeads. The as-prepared powders showed hollow sphere structure. Without adding PS, coral like yolk-shell structure was not formed owing to the fast drying of the droplets and the rapid decomposition of the metal salts. Therefore, in this study, PS nanobeads take roles of dispersing agent and pore generator. The prepared powders had majority of Ni metal phase due to carbothermal reduction reaction under a N_2 atmosphere during spray pyrolysis, as shown below XRD result. Therefore, the powders showed the low discharge capacity even though its cycle property was good, as shown below.

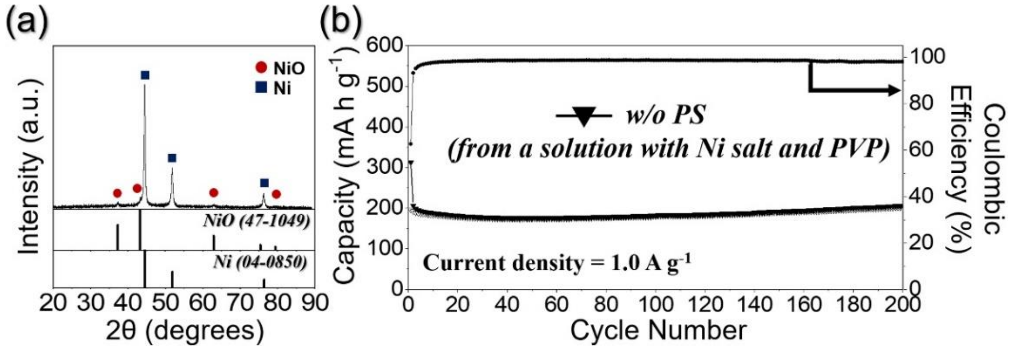

Fig. S11 a XRD pattern and **b** cycle property of the powders prepared from a solution containing Ni salt, PVP, and without PS nanobeads

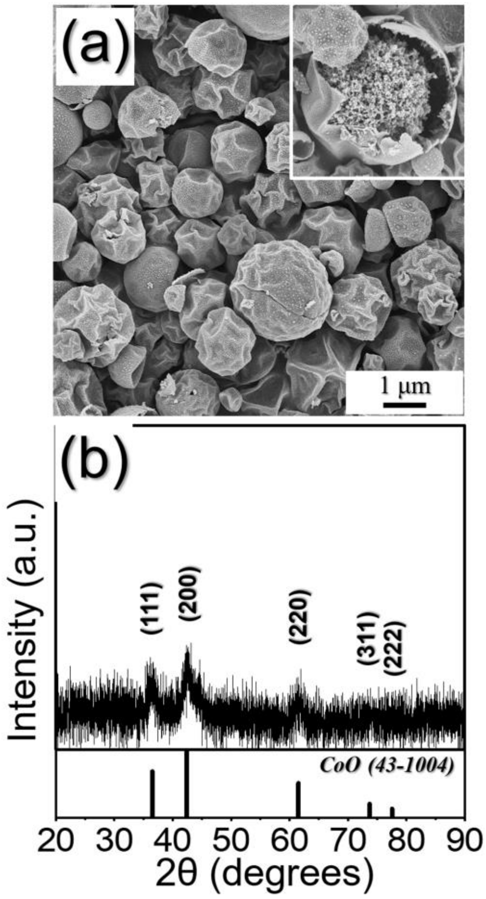

Fig. S12 a SEM image and **b** XRD pattern of the coral-like yolk-shell-structured CoO/C composite microspheres

Table S1 Electrochemical properties of the NiO materials with various morphologies as anode materials for LIBs

Materials	Voltage range (V)	Current Rate (mA g^{-1})	Initial C_{dis}/C_{cha} (mAh g^{-1})	Final discharge capacity (mAh g^{-1})	Cycle number	Refs.
CYS-NiO/C microsphere	***0.001-3***	***2,000<br>1,000***	***1,100/699<br>1,124/778***	***635<br>991***	***1,000<br>500***	***In this work***
NiO nanofibers	0.005-3	80	~1,280/~784	~583	100	[S1]
Porous NiO fiber	0.01-3	40	~1,100/696	~638	50	[S2]
NiO Nanowall	0.005-3	895	1,050/833	~638	85	[S3]
NiO@TiO_2 core-shell nanopowders	0.001-3	300	1,302/937	970	80	[S4]
NiO/RuO_2 composite carbon nanofibers	0.005-3	40	~650/~480	360	40	[S5]
NiO/MWCNT	0.05-3	50	1,084/720	800	50	[S6]
NiO nanoshafts	0.01-3	50	1,300/~860	410	30	[S7]
NiO microspheres	0.01-3	100	1,570/~1,060	100	30	[S8]
NiO hollow microspheres	0.02-3	100	1,100/620	560	45	[S9]
NiO-C nanocomposite	0.01-3	700	1,102/1,002	382	50	[S10]
NiO/graphene	0.02-3	100	~1,100/~730	646	35	[S11]
3D flower-like NiO	0.01-3	100	1,496/1,186	713	40	[S12]
NiO nanoflake arrays	0.01-3	100	900/~750	720	20	[S13]
Co-doped NiO nanoflake	0-3	100	1201/882	600	50	[S14]
Co-doped NiO nanoparticles	0-3	71.8	1,301/1,006	1,018	50	[S15]
NiO yolk-shell powders	0.001-3	700	~1,200/898	951	150	[S16]
Bamboo-like amorphous carbon nanotubes clad in ultrathin NiO nanosheets	0.01-3	800	1,377/936	1034	300	[S17]
Spherical and hollow-structured NiO aggregates	0.001-3	1,000	-	1,118	500	[52]
NiO nanofibers composed of hollow nanospheres	0.001-3	1,000	1,000/766	707	250	[58]
Hollow NiO nanooctahedrons	0.001-3	1,000	1175/-	1,234	150	[S18]
Egg shell-yolk NiO/C porous composites	0-3	100	1175/-	625	100	[S19]
NiO nano octahedron aggregates	0.01-3	143.6	1219/730	793	200	[S20]
Triple-shelled NiO hollow spheres	0.01-3	500	1,091/769	789	100	[S21]

Porous NiO hollow quasi-nanospheres	0.01-3	200	1149/850	~760	100	[S22]
Hierarchical NiO nanobelt film arrays	0.01-3	143.6	~1051/~795	~1035	70	[S23]
Flower-like NiO/RGO nanocomposites	0.01-3	100	~1492/~997	~702	100	[S24]
Porous NiO Nanorods	0.01	100	~743/-	~700	60	[S25]
Nanostructured NiO/C composite particles	0.01-3	70	~2398/-	~586	50	[S26]
3D hierarchical graphene@NiO@C composite	0.01-3	200	1490/1035	754	50	[S27]

Table S2 Fitted data obtained from the equivalent circuit for Nyquist plots

		R_e (Ω)	R_{ct} (Ω)
**Fresh cell**	CYS-NiO/C	7	314
	CYS-Ni/NiO/C	7	418
	Hollow NiO	7	374
**After 1th cycle**	CYS-NiO/C	6	15
	CYS-Ni/NiO/C	7	22
	Hollow NiO	6	25
**After 200th cycle**	CYS-NiO/C	5	18
	CYS-Ni/NiO/C	5	19
	Hollow NiO	6	223

References

[S1] V. Aravindan, P.S. Kumar, J. Sundaramurthy, W.C. Ling, S. Ramakrishna, S. Madhavi, Electrospun NiO nanofibers as high performance anode material for Li-ion batteries. J. Power Sources **227**, 284-290 (2013). https://doi.org/10.1016/j.jpowsour.2012.11.050

[S2] B. Wang, J.L. Cheng, Y.P. Wu, D. Wang, D.N. He, Porous NiO fibers prepared by electrospinning as high performance anode materials for lithium ion batteries. Electrochem. Commun. **23**, 5-8 (2012). https://doi.org/10.1016/j.elecom.2012.07.003

[S3] B. Varghese, M.V. Reddy, Z. Yanwu, C.S. Lit, T.C. Hoong et al., Fabrication of NiO nanowall electrodes for high performance lithium ion battery. Chem. Mater. **20**(10), 3360-3367 (2008). https://doi.org/10.1021/cm703512k

[S4] S.H. Choi, J.H. Lee, Y.C. Kang, One-pot rapid synthesis of core-shell structured NiO@TiO_2 nanopowders and their excellent electrochemical properties as anode materials for lithium ion batteries. Nanoscale **5**(24), 12645-12650 (2013). https://doi.org/10.1039/C3NR04406H

[S5] Y. Wu, R. Balakrishna, M.V. Reddy, A.S. Naira, B.V.R. Chowdari, S. Ramakrishna, Functional properties of electrospun NiO/RuO_2 composite carbon nanofibers. J. Alloys Compd. **517**, 69-74 (2012). https://doi.org/10.1016/j.jallcom.2011.12.019

[S6] C. Xu, J. Sun, L. Gao, Large scale synthesis of nickel oxide/multiwalled carbon nanotube composites by direct thermal decomposition and their

lithium storage properties. J. Power Sources **196**(11), 5138-5142 (2011). https://doi.org/10.1016/j.jpowsour.2011.02.001

[S7] L. Yuan, Z. P. Guo, K. Konstantinov, P. Munroe, H.K. Liu, Spherical clusters of NiO nanoshafts for lithium-ion battery anodes. Electrochem. Solid-State Lett. **9**(11), A524-A528 (2006). https://doi.org/10.1149/1.2345550

[S8] L. Liu, Y. Li, S. Yuan, M. Ge, M. Ren, C. Sun, Z. Zhou, Nanosheet-based NiO microspheres: controlled solvothermal synthesis and lithium storage performances. J. Phys. Chem. C **114**(1), 251-255 (2010). https://doi.org/10.1021/jp909014w

[S9] D. Xie, W. Yuan, Z. Dong, Q. Su, J. Zhang, G. Du, Facile synthesis of porous NiO hollow microspheres and its electrochemical lithium-storage performance. Electrochim. Acta **92**, 87-92 (2013). https://doi.org/10.1016/j.electacta.2013.01.008

[S10] M.M. Rahman, S.L. Chou, C. Zhong, J.Z. Wang, D. Wexler, H.K. Liu, Spray pyrolyzed NiO-C nanocomposite as an anode material for the lithium-ion battery with enhanced capacity retention. Solid State Ionics **180**(40), 1646-1651 (2010). https://doi.org/10.1016/j.ssi.2009.10.018

[S11] Y.J. Mai, S.J. Shi, D. Zhang, Y. Lu, C.D. Gu, J.P. Tu, J. Power Sources **204**, 155-161 (2012). https://doi.org/10.1016/j.jpowsour.2011.12.038

[S12] Q. Li, Y. Chen, T. Yang, D. Lei, G. Zhang, L. Mei, L. Chen, Q. Li, T. Wang, Electrochim. Acta **90**, 80-89 (2013). https://doi.org/10.1016/j.electacta.2012.11.103

[S13] H. Wu, M. Xu, H. Wu, J. Xu, Y. Wang, Z. Peng, G. Zheng, Aligned NiO nanoflake arrays grown on copper as high capacity lithium-ion battery anodes. J. Mater. Chem. **22**(37), 19821-19825 (2012). https://doi.org/10.1039/C2JM34496C

[S14] Y.J. Mai, J.P. Tu, X.H. Xia, C.D. Gu, X.L. Wang, Co-doped NiO nanoflake arrays toward superior anode materials for lithium ion batteries. J. Power Sources **196**(15), 6388-6393 (2011). https://doi.org/10.1016/j.jpowsour.2011.03.089

[S15] T.V. Thi, A.K. Rai, J.H. Gim, J.K. Kim, High performance of Co-doped NiO nanoparticle anode material for rechargeable lithium ion batteries. J. Power Sources **292**, 23-30 (2015). https://doi.org/10.1016/j.jpowsour.2015.05.029

[S16] S.H. Choi, Y.C. Kang, Ultrafast synthesis of yolk-shell and cubic NiO nanopowders and application in lithium ion batteries. ACS Appl. Mater. Interfaces **6**(4), 2312-2316 (2014). https://doi.org/10.1021/am404232x

[S17] X. Xu, H. Tan, K. Xi, S. Ding, D. Yu et al., Bamboo-like amorphous carbon nanotubes clad in ultrathin nickel oxide nanosheets for lithium-ion battery electrodes with long cycle life. Carbon **84**, 491-499 (2015). https://doi.org/10.1016/j.carbon.2014.12.040

[S18] S.-K. Park, J.H. Choi, Y.C. Kang, Unique hollow NiO nanooctahedrons fabricated through the Kirkendall effect as anodes for enhanced lithium-ion storage. Chem. Eng. J. **354**, 327-334 (2018). https://doi.org/10.1016/j.cej.2018.08.018

[S19] G. Li, Y. Li, J. Chen, P. Zhao, D. Li, Y. Dong, L. Zhang, Synthesis and research of egg shell-yolk NiO/C porous composites as lithium-ion battery anode material. Electrochim. Acta **245**, 941-948 (2017). https://doi.org/10.1016/j.electacta.2017.06.039

[S20] C. Wang, Y. Zhao, D. Su, C. Ding, L. Wang, D. Yan, J. Li, H. Jin, Synthesis of NiO nano octahedron aggregates as high-performance anode materials for lithium ion batteries. Electrochim. Acta **231**, 272-278 (2017). https://doi.org/10.1016/j.electacta.2017.02.061

[S21] H. Li, H. Ma, M. Yang, B. Wang, H. Shao, L. Wang, R. Yu, D. Wang, Highly controlled synthesis of multi-shelled NiO hollow microspheres for enhanced lithium storage properties. Mater. Res. Bull. **87**, 224-229 (2017). https://doi.org/10.1016/j.materresbull.2016.12.005

[S22] J. Xu, H. Tang, T. Xu, D. Wu, Z. Shi, Y. Tian, X. Li, Porous NiO hollow quasi-nanospheres derived from a new metal-organic framework template as high-performance anode materials for lithium ion batteries. Ionics **23**(12), 3273-3280 (2017). https://doi.org/10.1007/s11581-017-2160-4

[S23] N. Hu, Z. Tang, P.K. Shen, Hierarchical NiO nanobelt film array as an anode for lithium-ion batteries with enhanced electrochemical performance. RSC Adv. **8**(47), 26589-26595 (2018). https://doi.org/10.1039/C8RA03599G

[S24] X. Li, L. Fan, X. Li, H. Shan, C. Chen, B.Yan, D. Xiong, D. Li, Enhanced anode performance of flower-like NiO/RGO nanocomposites for lithium-ion batteries. Mater. Chem. Phys. **217**, 547-552 (2018). https://doi.org/10.1016/j.matchemphys.2018.06.050

[S25] Q. Li, G. Huang, D. Yin, Y. Wu, L. Wang, Synthesis of Porous NiO Nanorods as High-Performance Anode Materials for Lithium-Ion Batteries. Part. Part. Syst. Charact. **33**(10), 764-770 (2016). https://doi.org/10.1002/ppsc.201600084

[S26] L. Zhang, J. Mu, Z. Wang, G. Li, Y. Zhang, Y. He, One-pot synthesis of NiO/C composite nanoparticles as anode materials for lithium-ion batteries. J. Alloy. Compd. **671**, 60-65 (2016). https://doi.org/10.1016/j.jallcom.2016.02.038

[S27] X. Wang, L. Zhang, Z. Zhang, A. Yu, P. Wu, Growth of 3D hierarchical porous NiO@ carbon nanoflakes on graphene sheets for high-performance lithium-ion batteries. Phys. Chem. Chem. Phys. **18**(5), 3893-3899 (2016). https://doi.org/10.1039/C5CP06903C
